# Job vacancy at the European Centre for Disease Prevention and Control (ECDC)

**DOI:** 10.2807/1560-7917.ES.2020.25.45.2011124

**Published:** 2020-11-12

**Authors:** Eurosurveillance editorial team

**Affiliations:** 1 European Centre for Disease Prevention and Control (ECDC), Stockholm, Sweden

The European Centre for Disease Prevention and Control (ECDC) invites applications for the following position:

 

**Scientific Officer Infectious Disease Epidemiology**

The application deadline expires on 9 December 2020. 

** **

For more information, please visit the ECDC website: 

https://ecdc.europa.eu/en/work-us/vacancies?f%5B0%5D=deadline_date%3A1

